# Towards task shifting? A comparison of the accuracy of acute trauma-radiograph reporting by medical officers and senior radiographers in an African hospital

**DOI:** 10.11604/pamj.2015.21.308.6937

**Published:** 2015-08-27

**Authors:** Johan du Plessis, Richard Pitcher

**Affiliations:** 1 Division of Radiodiagnosis, Department of Medical Imaging and Clinical Oncology, Faculty of Medicine and Health Sciences, Stellenbosch University, Cape Town, South Africa

**Keywords:** Task-shifting, role-extension, radiographer, trauma-radiograph reporting

## Abstract

**Introduction:**

Due to the universal shortage of radiologists, medical officers are largely responsible for acute trauma radiograph reporting in public sector healthcare facilities in well-resourced countries. In poorly-resourced countries, a shortage of medical officers results in most acute trauma radiographs being unreported. In the European Union (EU), experienced radiographers with no specific training have been shown to be more accurate than medical officers in trauma radiograph reporting, while EU radiographers who receive additional training can reach accuracies comparable to radiologists. In some EU countries, the role of the radiographer has been extended to include trauma reporting. However, there has been no study of the accuracy of trauma radiograph reporting by radiographers in Africa, where task-shifting could yield potentially greater benefits, due to shortages of both radiologists and medical officers. The aim of this study was therefore to compare the accuracy of acute trauma-radiograph reporting by medical officers and senior radiographers in an African setting

**Methods:**

A prospective study was conducted at a South African hospital from November 2013-April 2014. Medical officers and senior radiographers reported the same set of appendicular skeleton trauma radiographs. Reporting accuracy, sensitivity and specificity were calculated using a consultant radiologist's report as the reference standard. Differences were evaluated using the Mann-Whitney U test, with p < 0.05 significant

**Results:**

Senior radiographers achieved significantly higher reporting accuracy and sensitivity than medical officers (81.5% vs 67.8%, p = 0.002)

**Conclusion:**

Senior radiographers represent a potentially important resource for acute trauma-radiograph reporting in the public healthcare sector in Africa.

## Introduction

Due to the universal shortage of radiologists, medical officers are largely responsible for acute trauma radiograph reporting in public sector healthcare facilities in well-resourced countries [1–3]. The shortage of medical officers in sub-Saharan Africa (SSA) results in most acute trauma radiographs being unreported, particularly in the rural areas [3, 4]. Considerable training, commitment and experience is required to achieve acceptable accuracy in trauma-radiograph reporting [5–7]. Fractures of the appendicular skeleton remain the most commonly missed injuries [8–10] and errors in trauma radiograph reporting may have significant clinical impact [11–13].

In the European Union (EU), experienced radiographers with no specific training have been shown to be more accurate than medical officers in reporting trauma radiographs of the appendicular skeleton [14], while EU radiographers who receive additional training can reach accuracies comparable to radiologists [15–18]. There are thus increasing data from high-income countries that radiographers constitute an important potential resource to help address imaging service demands. Some EU countries have already extended the role of specifically trained radiographers to include trauma-radiograph reporting. Furthermore, high-income countries on other continents are exploring this task-shifting option [5, 16, 19].

In Africa, a shortage of both radiologists and medical officers exacerbates radiological service pressures currently encountered in high-income countries. EU countries in which task shifting for radiographers has already been implemented have 47-110 radiologists per million population [20, 21], compared to 0-3 public sector radiologists per million population in SSA countries [22–24]. In addition, EU countries have in excess of 100 medical officers, nurses and midwives per 10,000 population, compared to an average of 11.4 skilled healthcare personnel per 10,000 population in SSA. Africa also manages a disproportionately high percentage of the global burden of trauma, with injuries constituting an important cause of death and disability [25–28].

However, there has been very limited published work analysing the potential role of radiographers in addressing Africa's radiological reporting pressures. Although South Africa (SA) is a middle-income country with approximately 7.7 medical practitioners per 10,000 population [29], which is well above the World Health Organisation's benchmark for the minimum medical personnel required for basic health interventions [30], the country has major healthcare challenges. Half of all SA healthcare expenditure and two-thirds of all SA doctors are in the urban private sector, serving approximately 17% of the population. Some parts of rural SA have 14 times fewer doctors than the national average. The provision of healthcare in such areas is thus more comparable to that in low-income countries [31]. SA has adopted the District Health System (DHS) for delivery of comprehensive primary health care to its peoples. District Hospitals (DH's) form an integral part of this system and are staffed by medical officers who provide generalist services to in- and outpatients. All DH's have a 24-hour Accident and Emergency unit and are equipped with plain X-ray facilities [32]. Many rural DH's in SA are staffed by relatively junior medical officers on short-term contracts, while these same hospitals have a core of permanent-staff radiographers with many years of clinical experience [33]. A recent study showed that the acute fracture detection rate of experienced SA-trained radiographers with no specific post-graduate training in radiological reporting was comparable to that of EU radiographers with similar experience [34]. However, there has been no study comparing the acute fracture detection rate of SA medical officers with experienced SA radiographers. If experienced SA radiographers are shown to have higher trauma-radiograph reporting accuracy rates than SA medical officers, consideration could be given to additional training for senior SA radiographers, with a view to role extension, or task shifting [35], particularly in the rural areas [33, 36, 37]. Furthermore, such a finding would have potential implications for other African countries, given the critical shortage of medical officers across the continent [30, 31].

Task shifting involves the delegation of tasks from more- to less-specialized health care workers who can competently and safely perform the assigned tasks. As such, it represents a method of strengthening and expanding the health workforce. The WHO recommends that task-shifting be pursued wherever there is evidence that it is safe and cost-effective, with quality of care and patient safety being the guiding principles [35].

The aim of this study was therefore to compare the accuracy of acute trauma-radiograph reporting by medical officers and senior radiographers in an African setting.

## Methods

The study was undertaken from November 2013 through April 2014 in the Trauma and Emergency Unit of a 1386-bed public-sector hospital in the Western Cape Province of South Africa. Nine (n = 9) permanent-staff, SA-trained, public sector radiographers with more than ten years of clinical experience, and eight (n = 8) SA-trained, public sector medical officers employed on short-term contracts in the Trauma and Emergency Unit were recruited and reported the same set of forty (n = 40) digital trauma radiographs of the appendicular skeleton. Study radiographs were selected following a customized search of the Trauma and Emergency Unit's picture archive and communication system (PACS) and were representative of the Unit's appendicular skeleton plain-film imaging profile. Examinations with and without fractures were included. Thirty adult (n = 30) and ten (n = 10) paediatric images, including ten normal radiographs were selected. The consensus report of three consultant radiologists served as the reference standard for fracture identification. Participants were blinded to clinical details other than the history of recent trauma. All images were reported from a standard diagnostic-quality radiology workstation. Thirty seconds were allowed for the evaluation of each image, consistent with the “Rapid Reporting” component of the final Fellowship Examination of the College of Radiologists of the Colleges of Medicine of South Africa at the time. Participants were simply required to assess the presence or absence of a fracture. Data were collected on a customized MS Excel spreadsheet and analysed using Statistica v. 12 (2014). For each image, individual responses were stratified as true positive (TP), true negative (TN), false positive (FP) or false negative (FN). Overall accuracy, sensitivity and specificity were calculated for the two groups and expressed by means and standard deviations. Non-parametric T-tests were used to compare the two groups, with a 5% significance level. Sub-analyses were conducted for adult and paediatric patients. **Ethics statement:** The study was approved by the Health Research Ethics Committee of the Faculty of Medicine and Health Sciences of Stellenbosch University (Ref:S13/05/105) and the CEO of the public-sector hospital. Written, informed consent was obtained from all participants. Anonymity of individual results was assured through the assignment of unique study numbers.

## Results

All the participating public sector medical officers had less than three years of post-registration clinical experience. Experienced radiographers achieved significantly higher overall accuracy in acute appendicular skeleton fracture reporting (81.5% vs 67.8%, p = 0.002) (Table 1). The superior radiographer performance was a reflection of significantly higher sensitivity (86.3% vs 68.7%, p = 0.003), since the groups demonstrated similar specificity (65%, p = 0.942). Sub-analyses by patient age showed that radiographers achieved higher sensitivity in both adult (87.7% vs 66.8%, p = 0.002) and paediatric (83.3% vs 72.5%) reporting, although the latter did not achieve statistical significance (p = 0.167), due to the small paediatric sample size (n = 10). There was no significant difference in the specificity of the groups, although medical officers showed higher specificity in paediatric reporting.

**Table 1:** Statistical comparison

	Radiographers (%)	Medical officers (%)	*p*-value
**Accuracy**	81.5	67.8	0.002
SD	0.065	0.081	
**Adults and children**			
Sensitivity	86.3	68.7	0.003
Specificity	65.0	65.5	0.942
**Adults**			
Sensitivity	87.7	66.8	0.002
Specificity	72.2	68.7	0.74
**Children**			
Sensitivity	83.3	72.5	0.167
Specificity	38.8	50.0	0.67

## Discussion

To the best of our knowledge, this is the first study of its kind on the African continent. The results are strikingly similar to those from EU countries over the past two decades [14–18]. Experienced radiographers with no additional training in plain-film reporting have been shown to perform significantly better than junior doctors, consistently achieving above 80% overall accuracy in acute fracture detection. The fact that these findings have now been mirrored in an African setting has important ramifications for healthcare planning on the continent. These data can serve as a stimulus for further work in the domain of diagnostic imaging in Africa, since basic imaging is increasingly being viewed as an essential component of health care [3, 4].

Currently, there is a critical shortage of healthcare workers globally, and the demand is increasing, driven by population growth, longer life expectancy, a rise in chronic disease, the HIV pandemic and technological advances in healthcare [30]. It is estimated that at least 7.2 million additional skilled health workers are currently required to meet the WHO standard of 22.8 doctors, nurses and midwives per 10,000 population, for provision of basic health interventions globally. It is anticipated that this figure will reach 12.9 million in the next two decades [38, 39]. The shortage is most acute in SSA, which has 24% of the world's disease burden, 10% of the world's population, 3% of the world's healthcare workers and less than 1% of world health expenditure [40]. Thirty-one African countries do not meet the UN target of 22.8 doctors, nurses and midwives per 10,000 population [38, 39]. There is an urgent need for nations to embark on medium- and long-term health workforce planning [38, 39]. Such planning requires accurate data and should integrate all cadres of health worker [41]. Task shifting should be an integral part of this planning. It should not be seen as an emergency response to health worker shortages, but rather as a dynamic process that constantly assesses whether tasks within the job description of highly trained but scarce personnel can be performed by personnel that are in greater supply, but have either less training or narrowly focused training [30].

There is also growing realisation that some form of task shifting will be required at many levels of healthcare in the future, to facilitate more equitable access to services. The implementation of task shifting requires careful planning and inter-disciplinary professional collaboration, to ensure quality of care and sustainability [42]. Many countries have not fully considered how task shifting could affect the number and mix of health workers. Furthermore, many countries have professional regulations that prohibit the most productive and efficient use of health workers [43]. It is against this background that the findings of this study are considered particularly important. While radiographers in our study achieved reporting accuracies in excess of 80%, it is accepted that 95% accuracy is required for clinical practice [15]. On the strength of our findings, consideration should be given to a pilot project training a select group of experienced SA radiographers for plain-film trauma reporting, according to EU guidelines [44, 45]. Should reporting accuracies of 95% or higher be achieved, task shifting for such radiographers would be justified. The initial successful training of even a small number of experienced, rural-based SA radiographers has the potential to improve the country's existing trauma-radiograph reporting infrastructure. The findings of such a pilot project could inform further work in other African countries.

Our small sample size limited statistical power and may have masked significant differences in the paediatric group. Sample size also precluded analysis by body part. Additionally, this study could not be conducted in the clinical domain, since reporting is not included in the scope of practice of SA radiographers. By precluding clinical correlation with radiological findings, study design may have negatively impacted overall accuracy and contributed to relatively low specificity (true negatives) in both groups. Similarly, the 30 second time-limit imposed for reporting may have decreased overall reporting accuracy, since the clinical domain has no such time constraints.

Our finding that junior SA medical officers achieved only 67.8% accuracy for trauma radiograph reporting highlights just how difficult such reporting can be and underscores the training and experience required to achieve competence in this area of practice [5–7]. Of note, UK Casualty Officers achieved just 57.0% accuracy in a similar study [14]. Our results suggest that undergraduate radiology training in SA should be re-evaluated in an attempt to better equip medical graduates for accident and emergency unit clinical responsibilities in the first years of medical practice. We have also identified a need amongst recently qualified SA doctors for continuous professional development in plain X-ray interpretation.

## Conclusion

It is hoped that this study will serve as a stimulus for further critical evaluation of the role of senior radiographers in the provision of healthcare in Africa. We have shown that senior SA radiographers represent a potentially important resource for acute trauma-radiograph reporting in the public healthcare sector and recommend a pilot project, training a select group of such radiographers for plain-film trauma reporting. The results of such a project could inform further work in other parts of Africa. Furthermore, accurate data on the number and distribution of radiographers in African countries is required to facilitate future health workforce planning.
